# Effect of Silica Particle Size on Macrophage Inflammatory Responses

**DOI:** 10.1371/journal.pone.0092634

**Published:** 2014-03-28

**Authors:** Toshimasa Kusaka, Masafumi Nakayama, Kyohei Nakamura, Mai Ishimiya, Emi Furusawa, Kouetsu Ogasawara

**Affiliations:** 1 Department of Immunobiology, Institute of Development, Aging, and Cancer, Tohoku University, Sendai, Japan; 2 Frontier Research Institute for Interdisciplinary Sciences, Tohoku University, Sendai, Japan; 3 Department of Hematology and Rheumatology, Tohoku University Graduate School of Medicine, Sendai, Japan; Juntendo University School of Medicine, Japan

## Abstract

Amorphous silica particles, such as nanoparticles (<100 nm diameter particles), are used in a wide variety of products, including pharmaceuticals, paints, cosmetics, and food. Nevertheless, the immunotoxicity of these particles and the relationship between silica particle size and pro-inflammatory activity are not fully understood. In this study, we addressed the relationship between the size of amorphous silica (particle dose, diameter, number, and surface area) and the inflammatory activity (macrophage phagocytosis, inflammasome activation, IL-1β secretion, cell death and lung inflammation). Irrespective of diameter size, silica particles were efficiently internalized by mouse bone marrow-derived macrophages via an actin cytoskeleton-dependent pathway, and induced caspase-1, but not caspase-11, activation. Of note, 30 nm-1000 nm diameter silica particles induced lysosomal destabilization, cell death, and IL-1β secretion at markedly higher levels than did 3000 nm-10000 nm silica particles. Consistent with *in vitro* results, intra-tracheal administration of 30 nm silica particles into mice caused more severe lung inflammation than that of 3000 nm silica particles, as assessed by measurement of pro-inflammatory cytokines and neutrophil infiltration in bronchoalveolar lavage fluid of mice, and by the micro-computed tomography analysis. Taken together, these results suggest that silica particle size impacts immune responses, with submicron amorphous silica particles inducing higher inflammatory responses than silica particles over 1000 nm in size, which is ascribed not only to their ability to induce caspase-1 activation but also to their cytotoxicity.

## Introduction

Inhalation of silica (also known as silicon dioxide) causes silicosis, which is one of the most prevalent occupational diseases worldwide [1], [2]. Globally, silicosis kills thousands of people each year throughout the world [1], [2]. Silicosis is irreversible and the disease progresses even when exposure to silica stops [1]. Silica is divided into two forms, crystalline and amorphous. Although these two forms are composed of the same molecular element (SiO_2_), their physical properties are different. Crystalline silica, which is the major component of sand and rocks, is thought to cause more severe pulmonary inflammation than amorphous silica [1]. Because amorphous silica is considered to be much safer than crystalline silica, it is used in a wide variety of materials, such as pharmaceutical products, paints, cosmetics, and food. Moreover, with the development of nanotechnology, practical uses for amorphous silica nanoparticles (<100 nm diameter particles) are rapidly expanding because they have unique physicochemical properties and exert innovative functions [3]. Strikingly, several recent studies have shown that both amorphous silica and crystalline silica are toxic *in vitro* and *in vivo* [4]–[6]. The toxicity of silica nanoparticles continues to be a matter of debate. For instance, silica nanoparticles have been reported to be more inflammatory than microparticles [4], [7], [8], although other reports have shown that the inflammatory potential of nanoparticles are less than, or largely equivalent to, microparticles [9]–[11]. Thus, a detailed study investigating the relationship between silica particle size and toxicity is required.

Macrophages have been suggested to play a crucial role in the initiation of silicosis [1]. Macrophages internalize silica particles and produce pro-inflammatory cytokines such as interleukin (IL)-1β and IL-18 [1]. Given that IL-1β–deficient mice are protected from silica-induced lung inflammation [12], and that administration of exogenous recombinant IL-1β causes pulmonary inflammation and fibrosis [13], IL-1β appears to be a critical mediator of silicosis.

Secretion of IL-1β is tightly controlled, and requires at least two specific signals [14]. First, pathogen-associated molecular patterns (PAMPs) such as lipopolysaccharide (LPS) and lipoproteins stimulate Toll-like receptors, leading to production of pro-IL-1β. A second signal causes pro-IL-1β to be processed by active caspase-1 into mature IL-1β[1], [15], [16]. Silica particles activate the second signal but not the first signal in macrophages [1]. In PAMPs-primed macrophages, silica particles stimulate the assembly of a Nalp3 (also called NLRP3 or Cryopyrin)-containing multiprotein complex called the inflammasome, which leads to activation of caspase-1 [15], [16]. It is thus predicted that silica particle size-dependent induction of IL-1β secretion is correlated with the level of caspase-1 activity, but this relationship has not been explored.

In this study, we investigated the relationship between the diameter of amorphous silica particles (modulating the dose, particle numbers, and surface area) and ensuing macrophage inflammatory responses to these silica particles in primary mouse macrophages. Furthermore, we examined the *in vivo* immunotoxicity of silica particles of different diameters, using a mouse model of lung inflammation.

## Materials and Methods

### Ethics statement

The animal study was performed according to the protocols approved by the Institutional Committee for Use and Care of Laboratory Animals of Tohoku University, which was granted by Tohoku University Ethics Review Board (No. 2012AcA-069) and the Guide for Care and Use of Laboratory Animals published by the U.S. National Institutes of Health (NIH publication 85–23, revised 1996). All surgery was performed under anesthesia by isoflurane for animal (Intervet, Tokyo, Japan). For sampling the tissues, mice were sacrificed by cervical dislocation. All efforts were made to minimize animal suffering.

### Mice

C57BL/6N mice (5–6 weeks old) were purchased from Nippon CLEA (Japan). Mice were maintained under specific pathogen-free conditions and used according to the guidelines of the Institutional Animal Care and Use Committee established at Tohoku University.

### Silica particles

All amorphous silica particles used in this study were purchased from Micromod Partikeltechnologie GmbH (Rostock, Germany). For all *in vitro* studies, the recognition of silica particles by macrophages was synchronized by brief centrifugation of silica particles onto the macrophages.

### Bone marrow-derived macrophages (BMDMs)

C57BL/6N mouse BMDMs were grown in complete RPMI-1640 (RPMI-1640 supplemented with 10% fetal bovine serum, 100 U/mL penicillin, 100 μg/mL streptomycin, and 2 mM glutamine) containing 1/10 volume of CMG-1412 culture supernatant containing macrophage colony-stimulating factor (M-CSF) for 5 or 6 days.

### *In vitro* interleukin (IL)-1β secretion from BMDMs

BMDMs (1×10^5^/well) were seeded into 48-well plates and cultured overnight. Then cells were primed with 10 ng/mL lipopolysaccharide (LPS; List Biological Laboratories, Campbell, CA). Five hours later, cells were stimulated with the indicated dose and size of silica particles or with 1 mM ATP (Wako, Osaka, Japan) for four hours at 37°C. For some experiments, LPS-primed BMDMs were pretreated with 20 μM of cytochalasin D (Cyto D; Wako, Japan), or 200 nM of Bafilomycin A1 (Sigma-Aldrich, St Louis, MO) for 1 hour, and were then stimulated with silica particles for 4 hours. The amount of IL-1β in cell culture supernatants was measured by an enzyme-linked immunosorbent assay (ELISA) kit (eBioscience, San Diego, CA) according to the manufacturer’s instructions.

### Internalization of silica particles by BMDMs

This assay was performed as described previously [17]. Briefly, cells grown on glass coverslips were cultured with fluorescein isothiocyanate (FITC)-labeled silica particles (50 μg/mL) for 1 hour at 37°C, then washed with phosphate buffered saline (PBS), and fixed with 10% formalin in PBS for 15 min. Cells were stained with phycoerythrin (PE)-labeled anti-CD11b monoclonal antibody (mAb) (BioLegend, San Diego, CA). Images were acquired using a Carl Zeiss LSM510 confocal laser-scanning microscope.

### Lysosomal damage in BMDMs

BMDMs grown on glass coverslips were primed with LPS (10 ng/mL) for 6 hours, and were loaded with 10 kDa FITC-dextran (1 mg/mL; Sigma-Aldrich) and exposed to silica particles (0.3 mg/mL) or ATP (1 mM) for 2 hours. BMDMs were then washed with PBS, and fixed with 10% formalin in PBS for 15 min. Intracellular distribution of FITC-dextran was analyzed by Leica TCS SP8 confocal laser-scanning microscope. Regarding the quantification of lysosomal damage, LPS (10 ng/mL)-primed BMDMs were loaded with LysoTracker-Red (200 nM) and treated with silica particles (0.3 mg/mL) or ATP (1 mM) for 2 hours. Percentage of LysoTrakcer-negative cells was quantified by FACSCantoII (BD, Franklin Lakes, NJ).

### Immunoblot analysis

Immunoblot analysis of inflammasome activation was performed as described previously [18]. Briefly, BMDMs were seeded in six-well plates and cultured overnight. Cells were primed with LPS (10 ng/mL) for 6 hours at 37°C. After replacing the media with serum-free media containing recombinant human M-CSF (50 ng/mL; Peprotech Inc., Rocky Hill, NJ), cells were stimulated with 0.3 mg/mL of silica particles for two hours at 37°C. Culture supernatants and total cell lysates were pooled and then clarified by centrifugation. Proteins were precipitated with Strataclean Resin (Stratagene, La Jolla, CA) and detected with anti-mouse IL-1β Ab (R&D systems, Minneapolis, MN), anti-mouse caspase-1 Ab (Santa Cruz, CA), anti-mouse caspase-11 mAb (Santa Cruz), anti-mouse β-actin mAb (Bio Legend).

### Lactate dehydrogenase (LDH) release assay

LPS (10 ng/ml)-primed BMDMs were stimulated with the indicated silica particles for 2 hours at 37°C. The cytotoxicity of silica against BMDMs was addressed by measuring LDH activities in cell culture supernatants using CytoTox 96 (Promega, Madison, WI) according to the manufacture’s instructions.

### Mouse model of lung inflammation

Silica particles (25 mg/kg) in saline or the vehicle alone were injected intra-tracheally (i.t.) into C57BL/6N mouse. Six or 24 hours later, lung inflammation was analyzed by micro-computed tomography (CT) scan LaThetaTM LCT-200 (Hitachi-ALOKA, Tokyo, Japan). Then bronchoalveolar lavage fluid (BALF) was harvested from mice, and the concentrations of IL-1β (R&D systems), TNF-α (eBioscience), and IL-6 (eBioscience) in BALF were analyzed by ELISA. For neutrophil infiltration in BALF, cells were stained with allophycocyanin (APC)-anti-CD45 (BioLegend) and FITC-anti-Gr-1 (BioLegend), and analyzed with a FACSCantoII.

### Statistical analysis

Statistical analysis was performed using the unpaired two-tailed Student's t-test: *P < 0.05; **P < 0.01. All data are represented as the mean + S.D.

## Results

### Submicron amorphous silica particles are strong inducers of IL-1β secretion from macrophages

**Figure 1:**
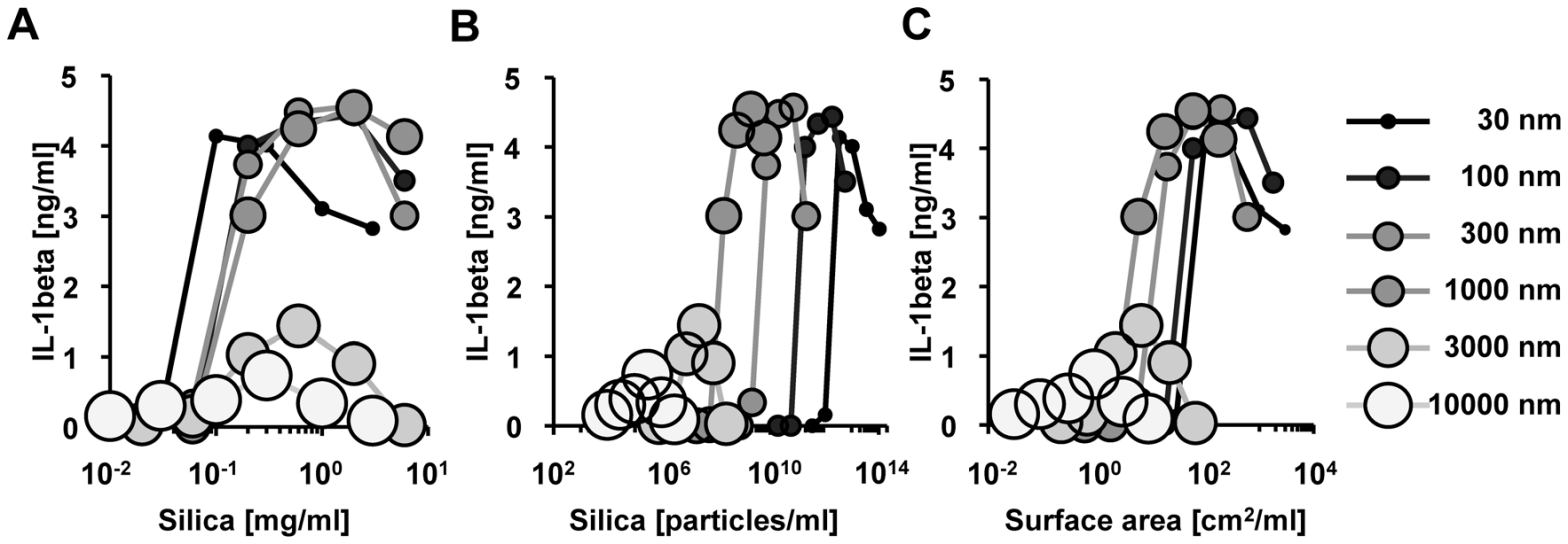
IL-1β secretion from bone marrow-derived macrophages (BMDMs) in response to different sizes of silica particles. C57BL/6 mouse BMDMs primed with LPS [10 ng/mL] were stimulated with silica particles of the indicated size for 4 hours. The amount of IL-1β in culture supernatants was measured by ELISA. The relationship between the amount of IL-1β secretion and (**A**) the dose, (**B**) the number, or (**C**) the surface area of the indicated silica particles is shown. S.D. was less than 10% of the mean of triplicates (not shown). Similar results were obtained in at least three independent experiments.

Several studies have shown that nanoparticles are more inflammatory than microparticles [4], [7], [8], [19], whereas others have shown the conflicting results [9]–[11]. To address this discrepancy, we carefully analyzed the relationship between silica particle size (dose, number, surface area, and diameter) and its pro-inflammatory properties. Specifically, we first stimulated LPS-primed BMDMs with amorphous silica particles of various sizes and measured IL-1β cytokine secretion. At around 0.1 mg/ml, 30 nm silica particles induced the higher amounts of IL-1β secretion from BMDMs than did the larger size of silica particles, however, at higher concentrations of 1 mg/mL, 100–1000 nm silica particles induced more IL-1β than did 30 nm silica particles (Figure 1A). Regarding the IL-1β secretion activity per particle number (Figure 1B) or per particle surface area (Figure 1C), 300 nm and 1000 nm silica were found to be stronger than 30 nm silica particles. When focused on the maximum IL-1β secretion level, 30 nm–1000 nm silica particles have the largely equivalent pro-inflammatory properties: any of those silica particles has ability to induce around 4 ng/ml of IL-1β secretion from BMDMs. Three thousand nm and 10000 nm silica has only one-forth of the pro-inflammatory property. Based on these results, we conclude that 1000 nm is the threshold diameter for maximum IL-1β secretion. Consistent with previous reports [15], [16], we did not detect IL-1β secretion from LPS-unprimed BMDMs following stimulation with any size silica particles (data not shown), suggesting that LPS-priming is required for IL-1β secretion by BMDMs. These results also illustrate that the silica particles used in this study were not contaminated with LPS. Together, these data indicate that submicron silica particles induce IL-1β secretion more effectively than larger silica particles.

### Actin polymerization is essential for the internalization of silica particles by BMDMs and subsequent IL-1β secretion

**Figure 2:**
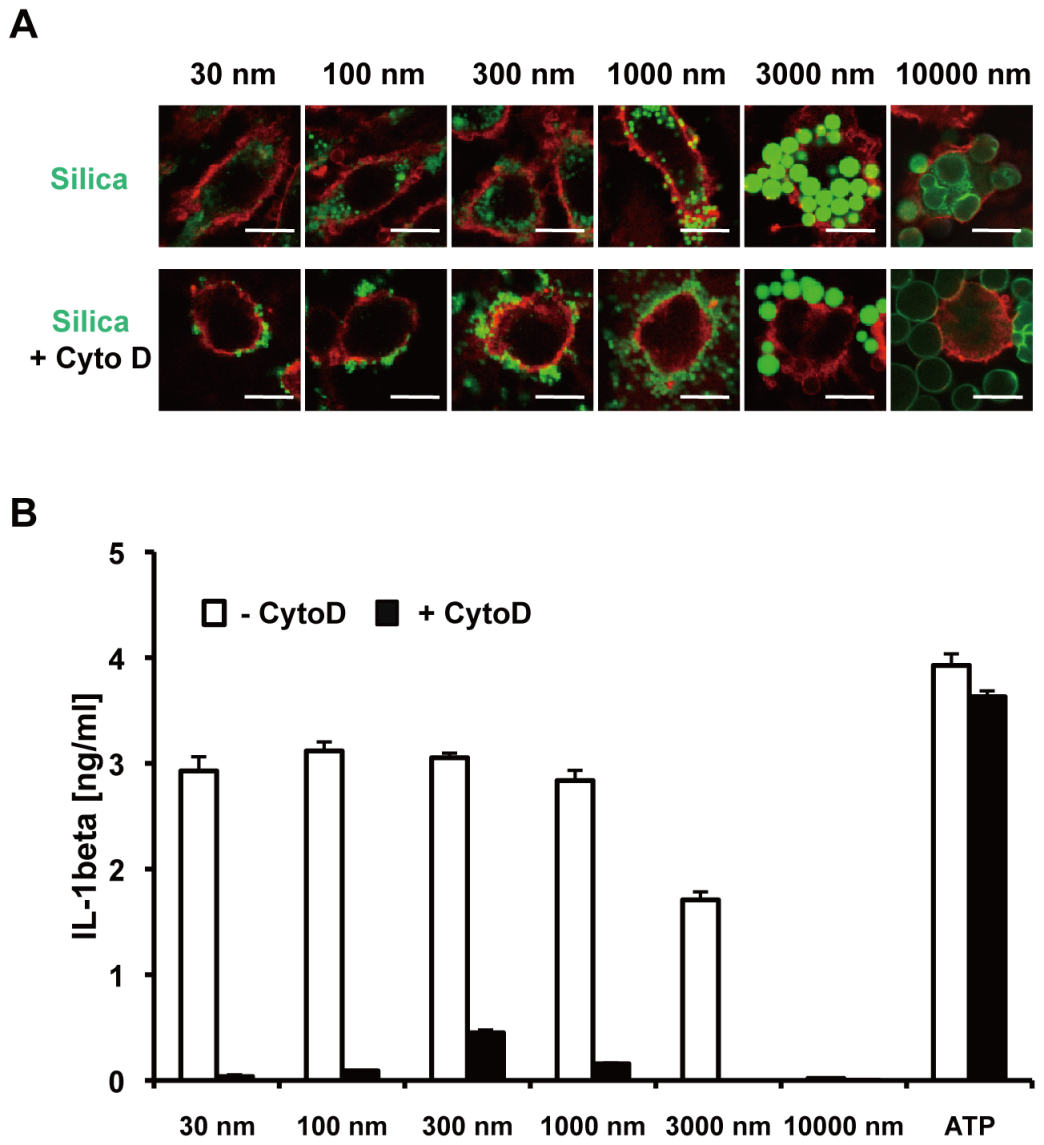
Silica particles of varying sizes are internalized by BMDMs via an actin cytoskeleton-dependent pathway. (**A**) BMDMs were incubated with (lower panels) or without (upper panels) cytochalasin D (Cyto D) [20 μM] for 1 hour, and were then stimulated with FITC-labeled silica particles [0.3 mg/mL] for 1 hour. After staining with PE-labeled anti-CD11b mAb, internalization of silica particles by BMDMs was analyzed by confocal microscopy. White bars indicate 10 microns. (**B**) LPS [10 ng/mL]-primed BMDMs were incubated with (black columns) or without (white columns) Cyto D [20 μM] for 1 hour, and then were stimulated with the indicated size silica particles [0.3 mg/mL] or ATP [1 mM] for 4 hours. The amount of IL-1β in culture supernatant was measured by ELISA. Data are indicated as the mean + S.D. Similar results were obtained in at least three independent experiments.

Since diameter size of silica particles strongly affects IL-1β secretion by macrophages, we next addressed whether particle size also affects the efficiency of particle internalization by macrophages. In this regard, we found that irrespective of particle size, BMDMs efficiently internalized silica, as indicated by FITC (green)-labeled silica particles localized inside PE (red)-labeled BMDMs (Figure 2A, and Figure S1). We further addressed whether the internalization is mediated via actin-cytoskeleton-dependent phagocytosis. Cyto D, an inhibitor of actin polymerization, dramatically inhibited the internalization of all sizes of silica particles tested, as indicated by silica particles localized outside BMDMs (Figure 2A and Figure S1). As a consequence, Cyto D reduced silica-induced IL-1β secretion (Figure 2B). Consistent with previous reports [16], [20], Cyto D did not inhibit ATP-stimulated IL-1β secretion, excluding the possibility that Cyto D inhibition of silica particle-induced IL-1β secretion was due to cellular cytotoxicity [21]. Together, these results suggest that silica particles of diverse sizes are efficiently internalized by BMDMs via cytoskeleton-dependent phagocytosis or endocytosis, and indicate that the internalization of particles is essential for IL-1β secretion.

### Submicron silica particles cause substantial lysosomal damage leading to IL-1β secretion

**Figure 3:**
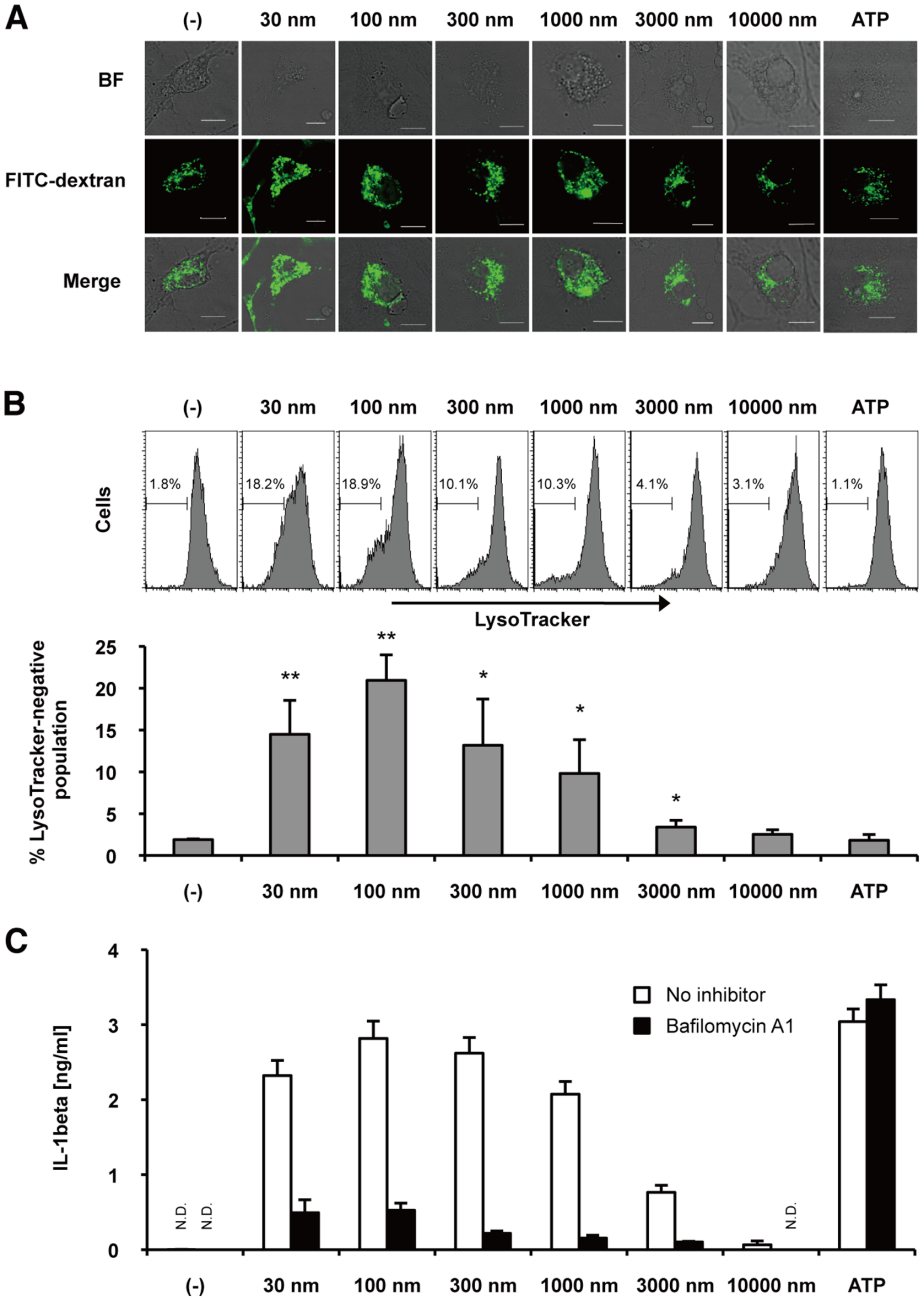
Lysosomal destabilization by silica particles of varying sizes. (**A**) LPS [10 ng/mL]-primed BMDMs were stimulated with FITC-dextran [1 mg/ml] and the indicated size of silica particles [0.3 mg/ml] or ATP [1 mM] for 2 hours. Intracellular distribution of FITC-dextran in BMDMs was analyzed by confocal microscopy. White bars indicate 10 microns. (**B**) LPS [10 ng/mL]-primed BMDMs were treated with LysoTracker-Red [200 nM], followed by silica particles of the indicated size [0.3 mg/ml] or ATP [1 mM] for 2 hours. Percentage of LysoTracker-negative population was quantified by flow cytometry. Columns represent mean + S.D. of triplicates. *P<0.05, **P<0.01 compared with (–) control. (**C**) LPS [10 ng/mL]-primed BMDMs were treated with (black columns) or without (white columns) bafilomycin A1 [200 nM] for 1 hour, and then were stimulated with the indicated size of silica particles [0.3 mg/mL] or ATP [1 mM] for 4 hours. The amount of IL-1β in culture supernatant was measured by ELISA. N.D., not detected. Data are indicated as the mean + S.D. Similar results were obtained in two (A) or three (B, C) independent experiments.

Internalized silica particles cause lysosomal damage [1], [9], [16], however, the relationship between silica particle size and lysosomal damage is not understood. Thus, we next addressed this issue. Ingested FITC-dextran was localized to small vesicular and tubular endosomes or lysosomes in macrophages [16], as shown in leftmost panels of Figure 3A. When BMDMs were exposed to 30 nm–3000 nm silica particles, some swollen lysosomes were observed in these cells (Figure 3A). Ten thousand nm silica-treated BMDMs did not show obvious swollen lysosomes. To quantify lysosomal damage, we analyzed BMDMs stained with fluorescent LysoTracker by flow cytometry. Consistent with confocal microscopy analysis, a significant population of BMDMs was LysoTracker-negative when exposed to 30 nm-3000 nm silica particles (Figure 3B), suggesting that these sizes of silica particles cause lysosomal damage. Of note, submicron silica caused more lysosomal damage than 3000 nm silica. To address the effect of lysosomal damage on IL-1β secretion, we blocked lysosomal acidification in BMDMs with bafilomycin A1, a specific inhibitor of vacuolar ATPase, and observed that bafilomycin A1 markedly suppressed silica-induced IL-1β secretion (Figure 3C). As expected, ATP treatment did not cause lysosomal damage (Figure 3A and B), and bafilomycin A1 did not suppress ATP-induced IL-1β secretion (Figure 3C). Taken together, these results suggest that submicron silica particles cause substantial lysosomal damage, which is associated with IL-1β secretion.

### Silica particles induce caspase-1 activation and IL-1β maturation, but not caspase-11 activation

**Figure 4:**
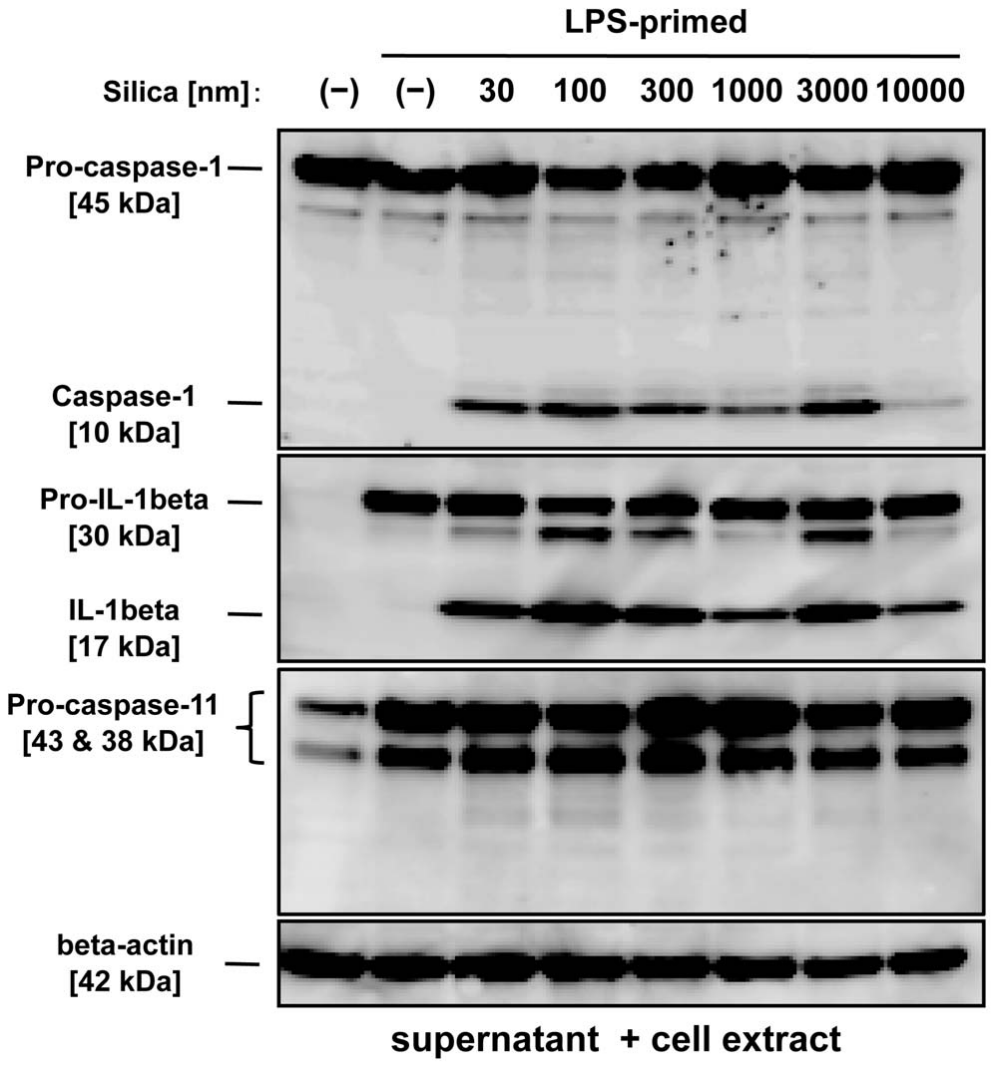
Inflammasome activation in BMDMs stimulated with silica particles of different sizes. LPS [10 ng/mL]-primed BMDMs were stimulated with the indicated sizes of silica [0.3 mg/mL] for 2 hours. The leftmost lane indicates LPS-unprimed and silica-untreated BMDMs. Maturation of caspase-1, IL-1β and caspase-11 in pooled supernatants and in cell extracts were analyzed by immunoblot. Anti-mouse β-actin antibody was used as a loading control. Similar results were obtained in at least three independent experiments.

Although internalized silica particles trigger IL-1β secretion in macrophages [9], the relationship between silica particle size and inflammasome activation is not fully understood. To this end, we next examined caspase-1 activation in BMDMs. We treated BMDMs with LPS for pro-IL-1β production¨followed by silica particles of various diameter sizes (Figure 4). Whereas the amount of IL-1β secretion induced by 30 nm-300 nm silica particles was much greater than that induced by 3000 nm silica particles (Figure 1), unexpectedly, there was no marked difference in caspase-1 activation or IL-1β maturation between BMDMs treated with 30 nm-300 nm silica and those treated with 3000 nm silica (Figure 4). These results indicate that silica size-dependent induction of IL-1β secretion is not correlated with its level of caspase-1 activity. In addition, we addressed whether silica particles activate caspase-11, which has been recently revived as an important inflammasome component [22]–[24]. However, we could not observe p26 mature form of caspase-11 in BMDMs stimulated with any size of silica particles (Figure 4). These results indicate that silica particles of widely varying sizes induce caspase-1 activation and IL-1β maturation, but not caspase-11 activation.

### Size-dependent cytotoxicity of silica particles toward BMDMs

**Figure 5:**
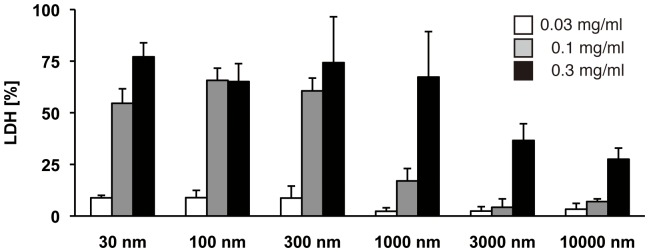
Cytotoxicity of different sizes of silica against BMDMs. BMDMs were stimulated with the indicated sizes of silica particles for 2(LDH) release. The percentage of LDH release was calculated as follows: 100 × experimental release/maximal (1% Triton-X-treated) release. Data are shown as the mean + S.D. of quadruplicate. Similar results were obtained in at least three independent experiments.

Because IL-1β secretion is associated with macrophage death [25], we hypothesized that silica size-dependent induction of IL-1β secretion may be ascribed to their cytotoxicity. Thus, we next performed the LDH release assay. Similar to IL-1β secretion (Figure 1), we observed that 30-1000 nm silica particles had higher cytotoxic activity toward BMDMs than did 3000 nm and 10000 nm silica particles (Figure 5). The cytotoxicity of silica particles was largely correlated with lysosomal damage-inducing activity (Figure 3). Taken together, these results suggest that the amount of silica-induced IL-1β secretion is influenced by not only caspase-1 activation, but also cytotoxic effects on macrophages.

### Intra-tracheal administration of 30 nm silica particles induces severe pulmonary inflammation in mice

**Figure 6:**
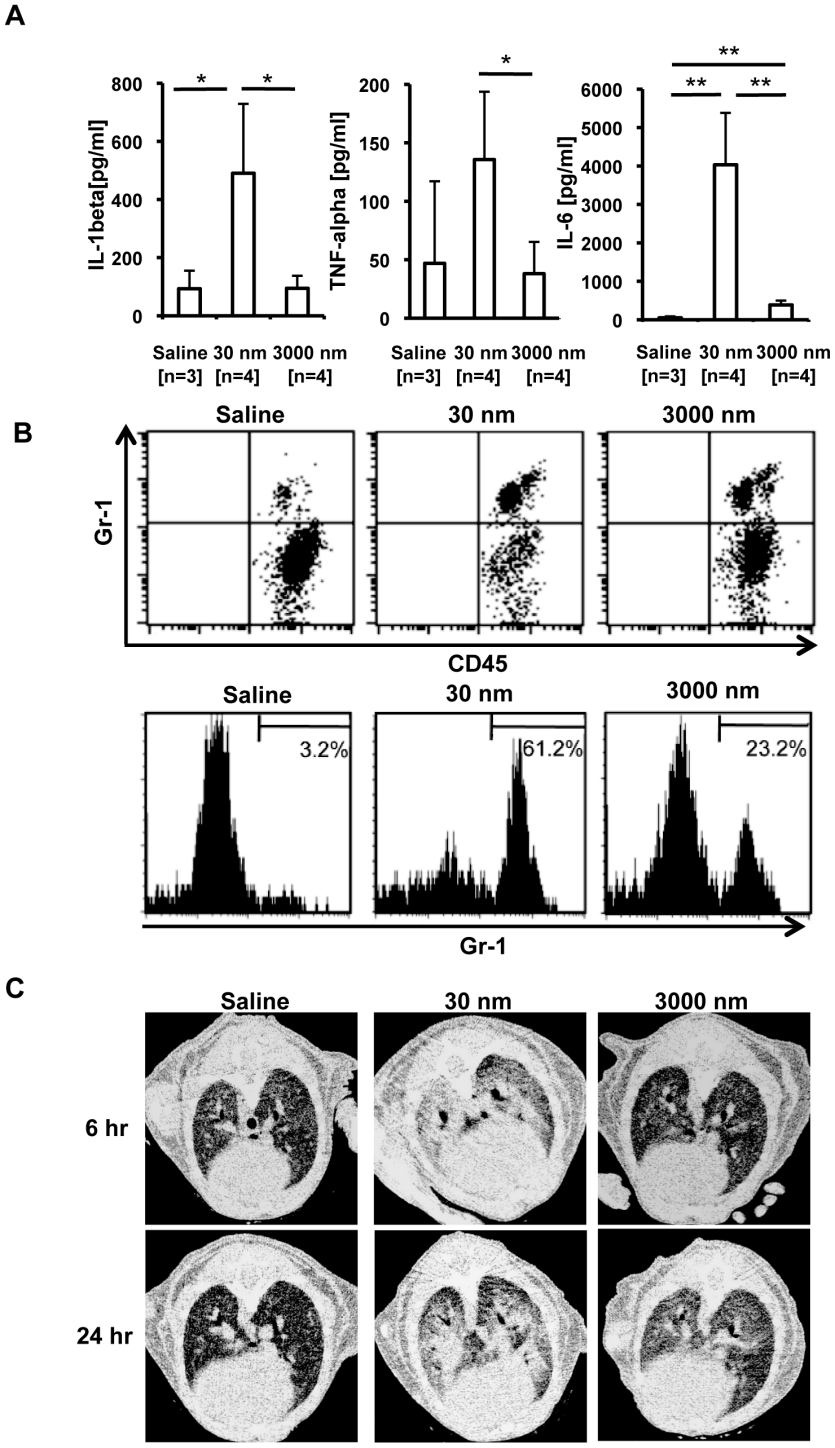
Silica-induced pulmonary inflammation. (**A**) C57BL/6 mice were injected intra-tracheally (i.t.) with saline (n = 3), 30 nm silica (n = 4) or 3000 nm silica (n = 4) [25 mg/kg]. Six hours later, bronchoalveolar lavage fluid (BALF) was harvested, and the amounts of IL-1β, TNF-α, and IL-6 were measured by ELISA. Data are indicated as the mean + S.D. *P < 0.05, **P < 0.01, two-tailed Student's t-test. Similar results were obtained in at least two independent experiments. (**B**) Cells in BALF harvested as described in panel (A) were stained with anti-Gr-1 mAb and anti-CD45 mAb, and analyzed by flow cytometry. Upper dot plots indicate Gr-1^+^ CD45^+^ cells and Gr-1^−^ CD45^+^ cells. Lower histograms indicate Gr-1 expression on CD45-positive cells. (**C**) Mice were treated as described in panel (A). Six or 24 hours later, lung inflammation was analyzed by micro-computed tomography. Similar results were obtained in at least two (A, B, C) independent experiments.

Although it was recently shown that nanoparticles cause massive lung inflammation [4], [5], [26], the relationship between silica particle size and lung inflammation has not been addressed in C57BL/6 mice. To this end, we administered 30 nm or 3000 nm silica particles i.t., and evaluated lung inflammation in these mice. Consistent with our *in vitro* results in BMDMs (Figure 1), 30 nm silica particles induced ∼5-fold greater levels of IL-1β in BALF than did 3000 nm particles (Figure 6A). LPS-priming was required for silica particle-induced IL-1β secretion in BMDMs (Figure 1), but not in mouse lungs, suggesting that pro-IL-1β exists in lungs of naïve mice housed under specific pathogen-free conditions. High concentrations of other pro-inflammatory cytokines such as TNF-α and IL-6 were also produced in BALF following administration of 30 nm silica particles (Figure 6A). These cytokines may be induced indirectly by IL-1β rather than directly by silica particles. Consistent with the high levels of pro-inflammatory cytokines, robust CD45^+^Gr-1^+^ neutrophils in the BALF were observed in mice treated with 30 nm silica particles (Figure 6B).

We further addressed silica particle-induced lung inflammation by micro-CT scanning. Figure 6C shows horizontal lung slices of mice treated i.t. with saline, 30 nm, or 3000 nm silica particles. Consistent with the highest level of pro-inflammatory cytokine production (Figure 6A) and the significant neutrophil infiltration in BALF (Figure 6B), aspiration of 30 nm silica particles caused severe pulmonary inflammation, as indicated by high intensity signals in the CT images. In contrast, 3000 nm silica particles caused mild pulmonary inflammation. Collectively, our results demonstrate that 30 nm silica particles cause more severe inflammation than 3000 nm silica particles both *in vitro* and *in vivo*.

## Discussion

In the present study, we demonstrated that submicron amorphous silica particles have greater inflammatory properties and cytotoxicity than silica particles measuring over 1000 nm, both *in vitro* and *in vivo*. Irrespective of diameter size, silica particles are internalized by macrophages via an actin cytoskeleton-dependent pathway, and induce inflammasome activation. The mechanism by which macrophages recognize silica particles is not fully understood. Although scavenger receptors such as SR-A and MARCO have been reported to recognize silica particles, the contribution of these scavenger receptors to the recognition of silica particles and subsequent IL-1β secretion is partial [27]–[29], suggesting the existence of unknown receptor(s) for silica particles. Furthermore, it remains unknown whether silica particles of different sizes are internalized by different receptors or by a single receptor. We are currently attempting to identify the receptor(s) for silica particles.

After internalization by macrophages, silica particles have been reported to disrupt lysosomes, resulting in the release of the lysosomal enzyme, cathepsin B, into the cytosol, and subsequently triggering Nalp3 inflammasome activation [16]. Likewise, several studies have shown that CA074ME, a cathepsin B-specific inhibitor, markedly inhibits the silica-induced IL-1β secretion [9], [19]. Contrarily, Dostert et al. have demonstrated that cathepsin B-deficient BMDMs normally respond to Nalp3 inflammasome activating danger signals [30], suggesting that cathespin B may not contribute to the Nalp3 inflammasome activation, or that genetic deletion of cathepsin B may result in increased another lysosomal enzyme(s) for the compensation, which may execute the inflammasome activation. In this study, we observed that submicron silica particles caused higher level of lysosomal destabilization than the larger silica particle, and that any size of silica-induced IL-1β secretion was markedly abrogated by CA074ME (data not shown) or bafilomycin A1, a vacuolar-type proton pump inhibitor. Although CA074ME may target some molecules distinct from cathepsin B, lysosomal damage is probably associated with IL-1β secretion. Given that lysosomal damage causes not only caspase-1-dependent pyroptosis [14] but also caspase-independent necrotic cell death [31], the lysosomal damage-mediated macrophage cell death may enhance mature IL-1β secretion. Our study using silica particles of various sizes suggests that, irrespective of diameter size, silica-induced IL-1β secretion may be mediated via a shared signaling pathway: cytoskeleton-dependent phagocytosis, lysosome disruption, caspase-1 activation, and cell death.

In order to assess the health and safety hazards of a wide variety of silica particles, a simple and accurate *in vitro* screening system must be established. Our *in vitro* study of IL-1β secretion from BMDMs reflected *in vivo* inflammatory responses measured in a mouse model of lung inflammation. Given that producing large numbers of BMDMs is a fairly simple process, our *in vitro* assay using BMDMs may represent a good first screen with which to predict the *in vivo* immunotoxicity of silica particles.

To assess the health effects of silica particles more precisely, toxicokinetic studies would also be required. A previous study demonstrated that in mice injected intravenously (i.v.) with gold particles, nanoparticles spread widely to various organs [32], suggesting that nanoparticles may cause multi-organ failure. In this regard, Nabeshi *et al.* recently reported that nanosilica can penetrate the skin barrier, and that mice injected i.v. with smaller silica particles show higher mortality [33], [34]. In this study, we chose an i.t. injection route because in the workplace, the risk of aspiration is much higher than the possibility of silica particles directly entering the blood stream. Consistent with a study using i.v. injection [34], we observed that many mice were moribund six hours after i.t. injection of 30 nm silica, but not 3000 nm silica (data not shown). It remains unclear whether 30 nm silica-induced lung inflammation is associated with the lethality. Thus, in addition to high inflammatory activity, smaller particles, including nanosilica, may be harmful in terms of their toxicokinetics.

When bioparticles such as bacteria and apoptotic cells are internalized by macrophages, in which these particles are digested, and cleared from our bodies [35]. In contrast to bioparticles, silica particles cannot be digested. Notably, even after phagocytosed by macropahges, intact silica particles would be eventually released from live and/or dying macrophages and likely stimulate neighboring macrophages. Presumably, forms of silica particles that cannot be excreted remain in the lung for an extended period of time, and may continually stimulate and disrupt macrophages, leading to chronic inflammation. Thus, highly inflammatory and unexcretable forms of silica would be most harmful. Overall, extensive studies of inflammation and toxicokinetics would be required for an assessment of the health risks of silica particles. Comprehensive analyses of the large data sets from “wet” experiments would make it possible to develop *in silico* predictions of silica toxicity.

## Supporting Information

Figure S1 **Cytochalasin D (Cyto D) inhibits uptake of silica particles by BMDMs: Wide-angle images of** **Figure 2A** **.** Cells were analyzed as described in Fig. 2A. In brief, internalization of silica particles by BMDMs was analyzed by confocal microscopy. The white bar represents 10 microns. Similar results were obtained in at least three independent experiments.
(TIF)
