# Supplementary figures and images for: Effect of Silica Particle Size on Macrophage Inflammatory Responses
Source: PLoS One. 2014 Mar 28;9(3):e92634. doi: 10.1371/journal.pone.0092634 (PMC3969333; doi:10.1371/journal.pone.0092634)

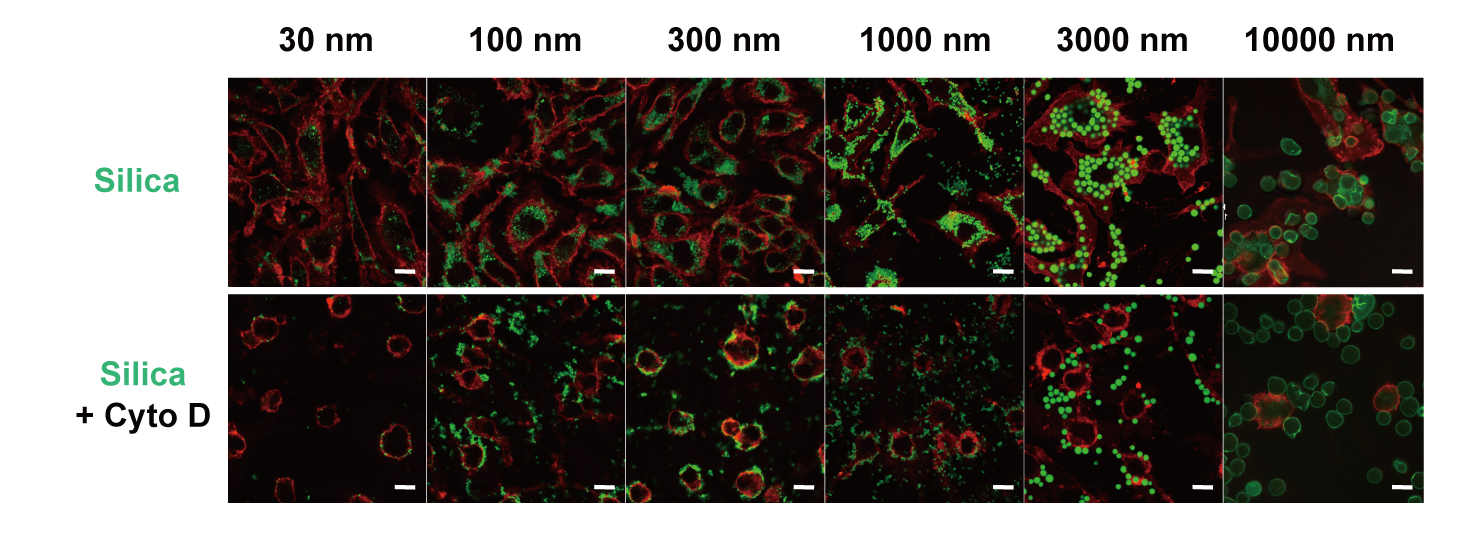

Supplement: Figure S1 — Cytochalasin D (Cyto D) inhibits uptake of silica particles by BMDMs: Wide-angle images of Figure 2A . Cells were analyzed as described in Fig. 2A. In brief, internalization of silica particles by BMDMs was analyzed by confocal microscopy. The white bar represents 10 microns. Similar results were obtained in at least three independent experiments. (TIF) [file pone.0092634.s001.tif]
